# Development of polarization modulator using MXene thin film

**DOI:** 10.1038/s41598-022-10768-x

**Published:** 2022-04-26

**Authors:** Zian Cheak Tiu, Sin Jin Tan, N. Yusoff, Harith Ahmad

**Affiliations:** 1 Faculty of Engineering and Quantity Surveying, INTI International University, 71800 Nilai, Negeri Sembilan Malaysia; 2 School of Engineering, UOW Malaysia KDU University College, 40150 Shah Alam, Selangor Malaysia; 3 Photonic Research Centre, University of Malaya, 50603 Kuala Lumpur, Malaysia

**Keywords:** Optics and photonics, Physics

## Abstract

In this work, polarization modulator utilizing MXene material, namely Nb_2_C is demonstrated. S band signal is injected into Nb_2_C thin film and is modulated by 1400 nm laser diode. A total of 39.81° of polarization rotation is attained when the pump power is increased to 223 mW. The rotation of light is due to thermo-optic effect. The efficiency of polarization modulator is calculated at 0.1974°/mW.

## Introduction

In the development of optical fiber communication system, first telecom window (800–900 nm) was obsoleted due to the nature of high propagation loss in single-mode fiber (SMF). Second window (O-band) was proposed for optical fiber communication system with the strength of near zero dispersion characteristic in optical fiber. However, there are no high-quality fiber amplifier in O-band region. Praseodymium had been practically proven as a rare earth element emitting in O-band region, but the emission efficiency is not sufficient to support the optical communication needs. As a result, third telecom window (C-band) have been proposed and widely used in optical communication system. Owing to the strong emission of Erbium-doped fiber amplifier and low propagation loss characteristic in SMF, C-band has been widely used for long-haul optical communication system. With the increasing demand of data transmission rate, third window has expanded from C-band to C+L-band^1–3^. The expansion to L-band does not face any major scientific difficulty, as L-band is still under the Erbium emission range. C+L band has proven its feasibility for high-speed transoceanic optical fiber communication in year 2013^4,5^. Since then, the core axis of high-speed optical fiber communication focuses on C+L-band^6^. Come to the era of Fifth Generation (5G) telecommunication system, the demand of bandwidth and data transmission rate are continuously increasing. C+L-band bandwidth will eventually reach the saturation point. Therefore, telecommunication industries are venturing into the expansion of optical communication bandwidth, and S-band region has been highlighted as highly feasible band to incorporate into C+L-band system^7,8^. Erbium-doped fiber amplifier may not be a suitable candidate to support the S-band region, but the development of ultra-wideband amplification technologies such as semiconductor optical amplifiers and thulium-doped fiber amplifiers are timely to support the development of S+C+L-band.

To achieve high speed and stable optical communication, it is crucial to have full control of the light propagation characteristics. Among the light characteristics, polarization play an important role, especially in long-haul optical communication system. Polarization issues can be visualized as polarization mode dispersion (PMD) in optical fiber, polarization dependent gain (PDG) in active optical devices, and polarization dependent loss (PDL) in passive optical components. Tangibly, polarization modulation is a critical technology to remedy polarization impairment in optical communication system. Mechanical polarization control techniques such as the combination of Quarter-wave-plate (QWP) and Half-wave-plate (HWP), QWP-HWP-QWP or 3-paddles polarization controller are not feasible to integrate into optical fiber system nor to provide precise control of polarization state. On the other hand, electronic-driven polarization control techniques such as LiNbO_3_ based polarization modulators are comparatively more feasible to integrate into optical communication system^9^. However, the high implementation cost, bulkiness in size and complex design are restricting the technology to expand from laboratory usage to industrial telecommunication system. The imperfection of mechanical and electronic-driven techniques hasten the development of all-optical polarization modulation technique to remedy the polarization impairment issues.

With the tremendously growth of two-dimensional (2D) materials, integration of 2D materials in fiber optic system to achieve all-optical modulation becoming core axis in optical modulation research area. Since the discovery of graphene, many types of 2D materials have evolved, such as transition metal dichalcogenides (TMDs), topological insulators (TIs), black phosphorus (BP) and MXene^10–13^. These 2D materials have proven their potential in optical modulation. For instance, MoWS_2_-rGO is used as phase shifter^14^; Bi_2_Te_3_ is used as temporal and amplitude modulators^15^; MoS_2_ is used as polarization modulator^16^; and MXene is employed for optical wavelength conversion^17^. The success of 2D materials in optical modulation are attribute to the exceptional optical properties, including strong nonlinearity, fast recovery time and broadband saturable absorption. Furthermore, the optical application of 2D materials are not confined in optical modulation, but also contributing in optical photodetection^18,19^, ultrafast technology^20–25^ and optical transistors^26,27^. Undoubtedly, 2D materials are highly potential to revolve the development in all-optical technologies.

In this work, Niobium Carbide (Nb_2_C), a family member of MXene, is processed into a thin film and integrated into all-optical system to function as polarization modulator in S-band region. The fundamental concept of this work is to induce thermo-optic effect on Nb_2_C thin film by pumping high intensity continuous wave (CW) laser. The accumulation of heat in Nb_2_C thin film alters the refractive index of itself, and thus modulate the polarization state of the signal light that propagate through the thin film. Throughout the pump power range of 0–223 mW, the proposed polarization modulator has achieved a rotation of 39.81°, which gives the modulation resolution of 0.1974°/mW. To the best of authors knowledge, this is the first all-optical polarization modulation in S-band region using MXene as modulator.

## Fabrication and characterization of Nb_2_C

### Fabrication of Nb_2_C/PVA film

The solution casting technique was utilized to fabricate the Nb_2_C/PVA film which follows the same procedure as reported in our previous work^28^. Initially, 50 mg of Nb_2_C powder was mixed into 10 mL of the IPA solution using a Hielscher UP200Ht Handheld Ultrasonic Homogenizer probe-type sonicator. The mixture was undergoing a sonication process for 4 h to obtain a homogenous suspension. The sonicator was set at 5 s on and off pulse sequence under the operating power of 80 W. In the next step, the suspension was centrifuged at 4000 rpm for 10 min to remove the undissolved powder and collecting the supernatant for further used. In order to fabricate the film, 5 mL of Nb_2_C solution was added into a glass beaker which consist of 5 mL of the PVA solution (10 mg/mL concentration) under constant stirring at 70 °C. After 1 h of stirring, the obtained sample was dried in an oven at the temperature of 60 °C for several hours. At last, the fabricated Nb_2_C/PVA film was obtained by peeling off the film from the petri dish and kept in a clean container for storage until further used as SA material. Owing to the low absorption loss in S-band region, the PVA is chosen as to form the thin film with Nb_2_C in this work. The Nb_2_C MXene powder was received from 2D Semiconductors and the polyvinyl alcohol (PVA) powder (MW ~ 31,000) and isopropyl alcohol (IPA) (~ 99.7%) solution were purchased from Sigma Aldrich. All reagents were used as received without purification.

### Characterization of Nb_2_C MXene

**Figure 1:**
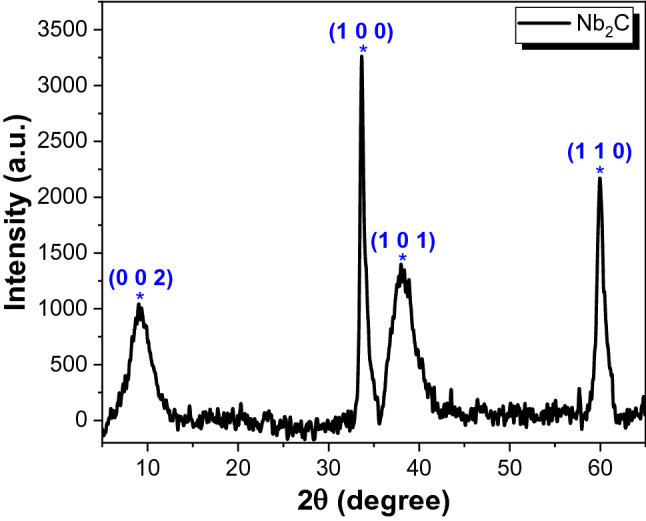
XRD pattern for Nb_2_C MXene in powder form.

The crystalline structure and phase of Nb_2_C MXene was examined by a Malvern Panalytical Empyrean X-ray Diffraction (XRD) operated using Cu Kα radiation. Figure 1 shows the XRD pattern for Nb_2_C MXene scanned from the 2θ of 5°–70°. A broadened diffraction peak located at about 9.04° was associated to the (0 0 2) plane of the Nb_2_C MXene. It has been reported that this peak was arise by cause of the interruption on the Nb-Al bond which lead to the increment of the interlayer spacing. The appearance of this diffraction peak has evident the successful of removing the Al layers upon the etching process and hence forming the Nb_2_C MXene^29^. Moreover, three more diffraction peaks can be observed at 33.68°, 38.01°, and 60.00° which ascribed to the (1 0 0), (1 0 1), and (1 1 0) plane of the hexagonal Nb_2_C MXene (JCPDS file 00-015-0127)^30^. The absence of other impurity peak indicates the high purity of the Nb_2_C MXene sample.

**Figure 2:**
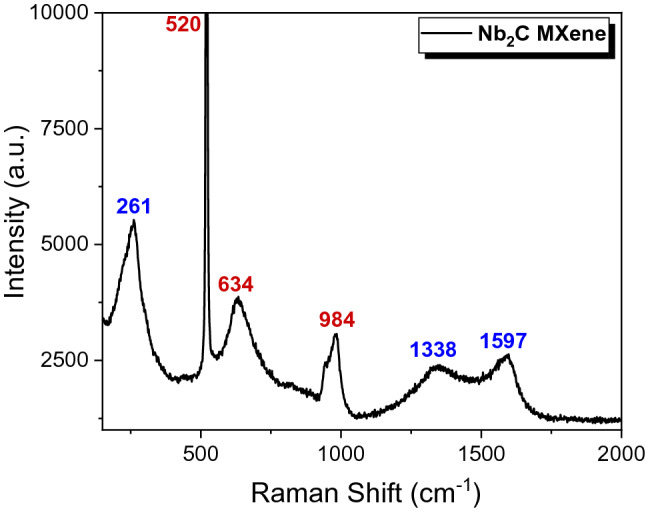
Raman spectrum of Nb_2_C MXene.

The Raman spectrum was obtained by an inVia Renishaw Raman spectrometer with a 514 nm laser as the excitation source and the result was presented in Fig. 2. According to the obtained result, a peak located at 261 cm^−1^ was observed which associated to the A_1g_ mode that arises due to the symmetrical out-of-plane vibrations of Nb and C atoms^31^. Besides that, two more characteristics peaks were appeared at the Raman shift of 1338 and 1597 cm^−1^ that can be indexed to the D and G bands of carbon species, respectively, in which carbon is one of the main constituent for Nb_2_C MXene sample. The D band refers to the carbon disordered structure of the sp^3^ hybrid carbon meanwhile the G band is assigns to the graphitic band or sp^2^ carbon atom^32,33^. It is worth noting that the remaining peaks located at 520, 634, and 984 cm^−1^ corresponds to the characteristic peaks of silicon (Si) substrate^34^.

**Figure 3:**
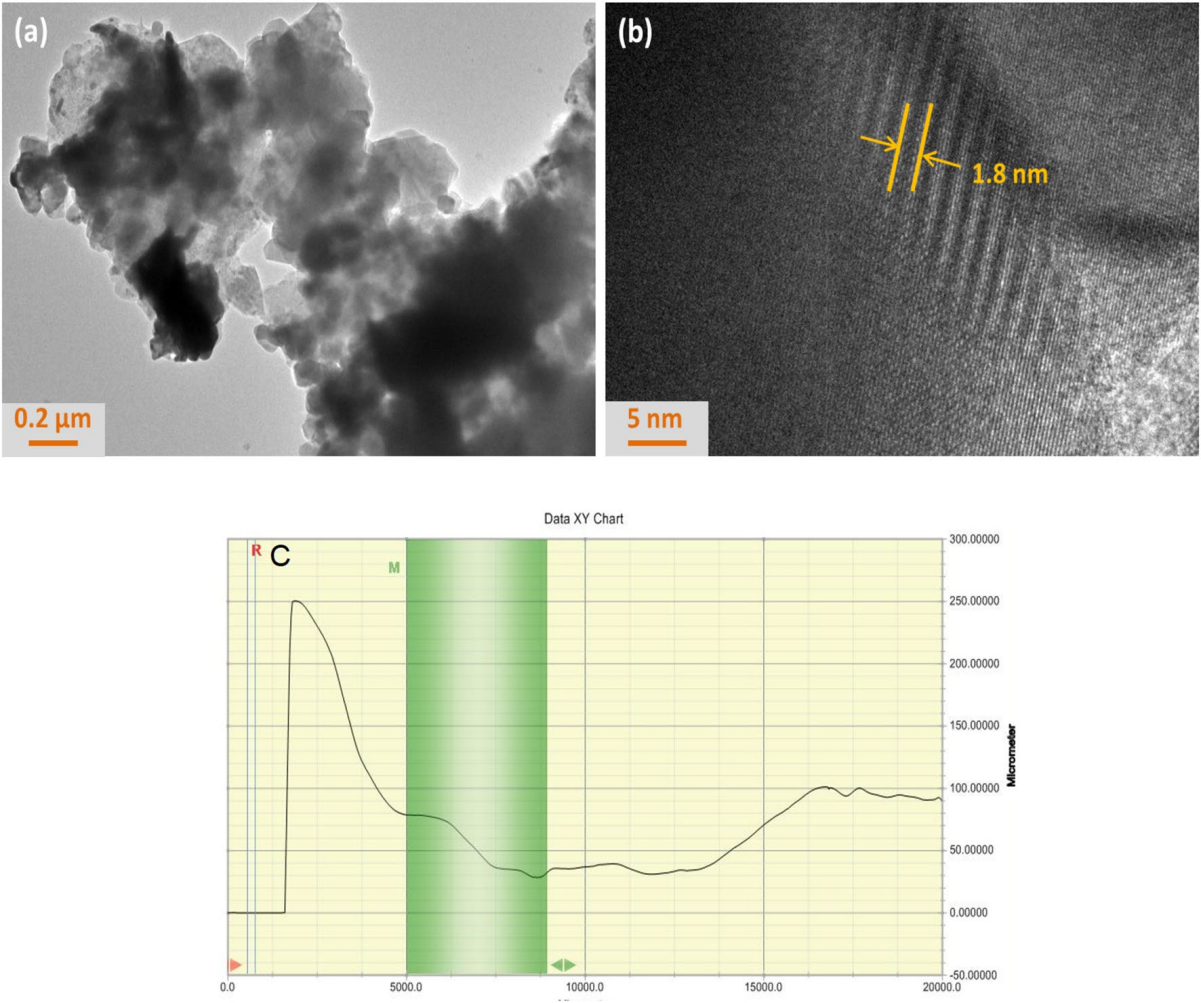
Morphology of HRTEM images of Nb_2_C MXene (**a**) HRTEM image of Nb_2_C MXene at low magnification (**b**) HRTEM image of Nb_2_C MXene at low magnification and (**c**) surface profiler measurement.

The high resolution transmission electron microscope (JEM 2100-F HRTEM) was utilized to investigate the morphology of the Nb_2_C MXene. Initially, the Nb_2_C solution was diluted by using ethanol as the solvent and the solution was sonicated for 20 min before drop casted onto the copper mesh for testing. Figure 3 depicts the HRTEM images of Nb_2_C captured at different magnification. As can be seen in Fig. 3a, Nb_2_C exhibits the layered feature with irregular shapes and sizes and the self-stacking occurs between the nanosheet layers. The light grey color indicates the thin area of the sample whereas the black color implies the much thicker area. Based on the HRTEM image of Nb_2_C obtained at high magnification, a well-defined hexagonal lattice can be noticed in Fig. 3b, which signifies the high crystallinity of Nb_2_C MXene. The interlayer spacing of Nb_2_C MXene was measured to be about 1.8 nm, thus verifies that the Nb_2_C samples consist of multiple layers. The thickness of the Nb_2_C-PVA film was measured using a Dektak D150 surface profiler after the produced film was placed on a glass slide. The Nb_2_C-PVA film was found to have a thickness of 52.5 μm as shown in Fig. 3c. The insertion loss of Nb_2_C/PVA thin film was measured about 3.2 dB at wavelength of 1500 nm.

## Experimental setup

**Figure 4:**
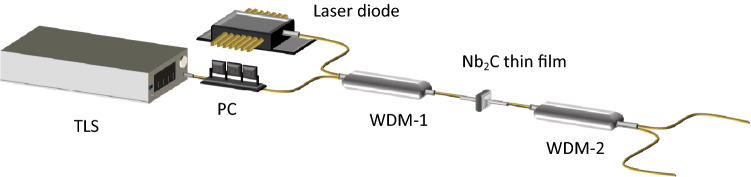
Experimental setup for polarization modulator utilizing Nb_2_C thin film.

Figure 4 shows the proposed setup for polarization modulator. A 1400 nm laser diode is used as a pump source. Santec TLS 550 tunable laser source (TLS) provides an input signal to the polarization modulator. TLS is set to 1500 nm and 0 dBm. A polarization controller (PC) is connected to TLS where it is functioning to rotate the light from TLS into polarized light. The 1400/1500 Wavelength Division Multiplexer (WDM-1) multiplexes both signals from laser diode and TLS. Nb_2_C thin film is cut into 2 × 2 mm and sandwiched in between two fiber ferrules. Another 1400/1500 (WDM-2) is located after Nb_2_C thin film to guide the input signal into Thorlab PAX1000 polarimeter for analysis.

## Results and discussion

**Figure 5:**
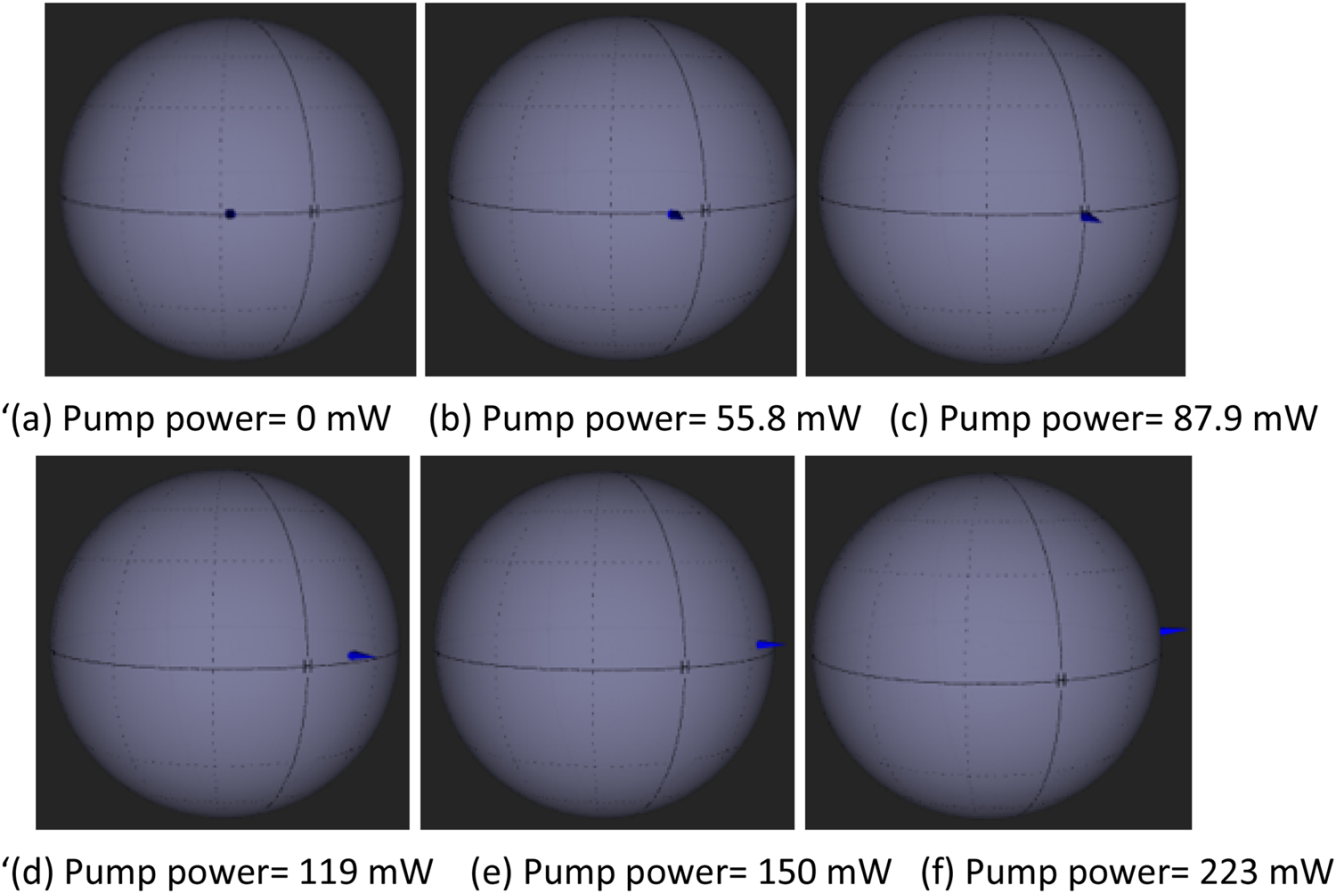
Evolution of Light polarization state rotation at various pump powers in 3D spherical view (**a**) 0 mW (**b**) 55.8 mW (**c**) 87.9 mW (**d**) 119 mW (**e**) 150 mW (**f**) 223 mW.

**Figure 6:**
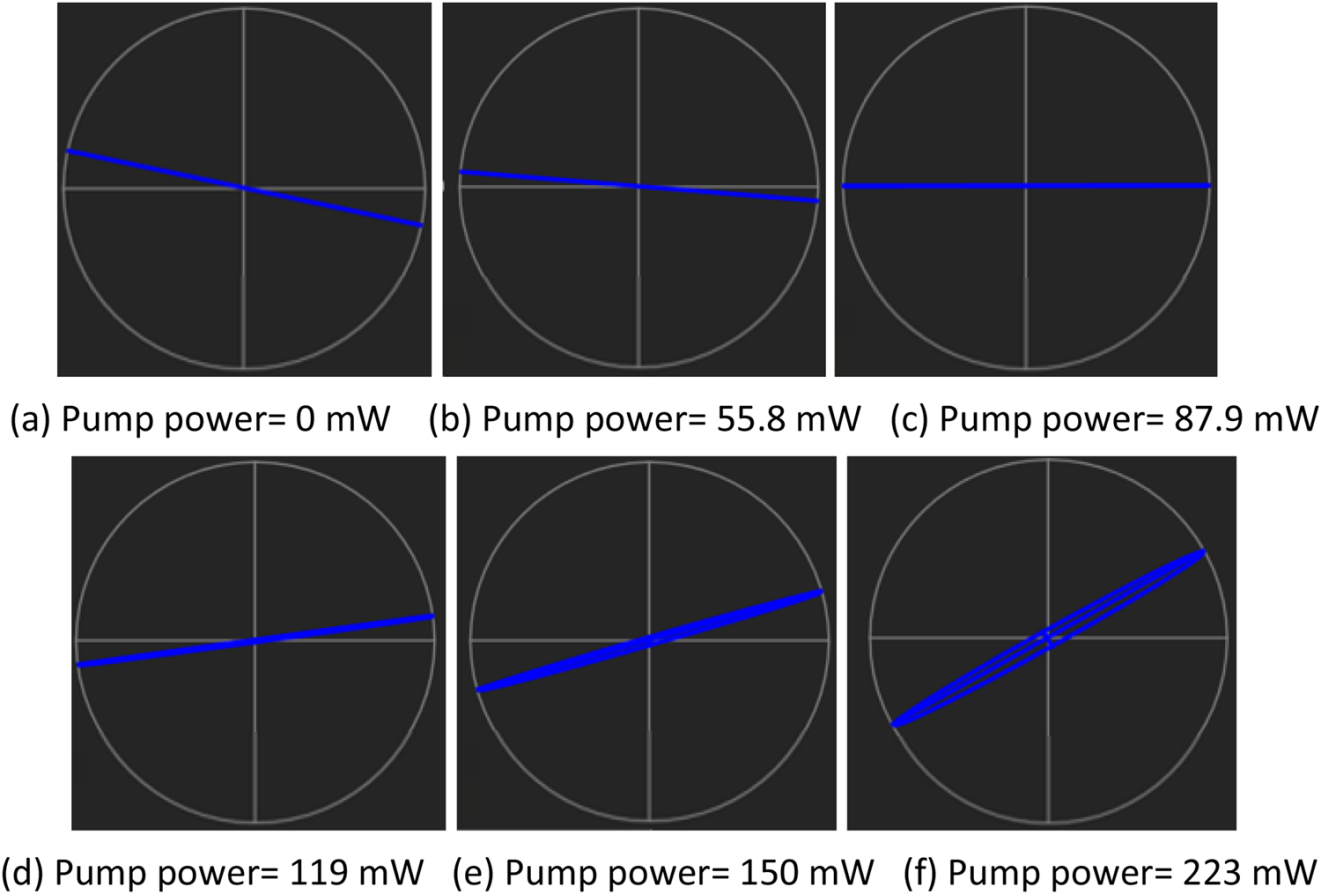
Evolution of light polarization state rotation at various pump powers in 2D view (**a**) 0 mW (**b**) 55.8 mW (**c**) 87.9 mW (**d**) 119 mW (**e**) 150 mW (**f**) 223 mW.

Tunable Laser Source (TLS) serves as an input signal to the polarization modulator. It is fixed at wavelength of 1500 nm and output power of 0 dBm. The orientation of PC is adjusted to change the TLS signal to a linearly polarized input signal, with − 11.94° azimuth and ellipticity of 0.13°. The linearly polarized input signal is as shown in Fig. 5. The measured output power is − 4.4 dBm. This indicates that the total loss of − 4.4 dBm is contributed by the thin film, connector, WDM and light polarization loss. The 1400 nm pump power is gradually increased up to the maximum available pump power at 223 mW. Figure 5 describes the polarization state rotation of light observed at polarimeter with the increment of pump power. The blue indicator on the spherical plot illustrates the polarization state of signal in 3 Dimension (3D) view. The blue indicator is moving in a counterclockwise direction with the increment of pump power. At 87.9 mW pump power, light azimuth is recorded at 0.08°. When the pump power is further raised to 119 mW, the indicator crosses to 7.67° and finally to 27.87° at pump power of 223 mW. As can be seen from the Fig. 5a–f, the indicator moves steadily near the horizontal axis, throughout the increment of pump power, signifying there is a continuous azimuth rotation. Figure 6 provides a better illustration on the rotation of light azimuth and ellipticity. The initial polarized light from TLS is represented by a straight line, tilted at an azimuth angle of − 11.94° and ellipticity of 0.13°. The light experienced counterclockwise rotation throughout the increment of pump power. At 87.9 mW, the light polarization is horizontally aligned with azimuth angle 0.08° while its ellipticity of 0.06°. At 223 mW, the measured azimuth and ellipticity is 27.87° and 3.77°.

**Figure 7:**
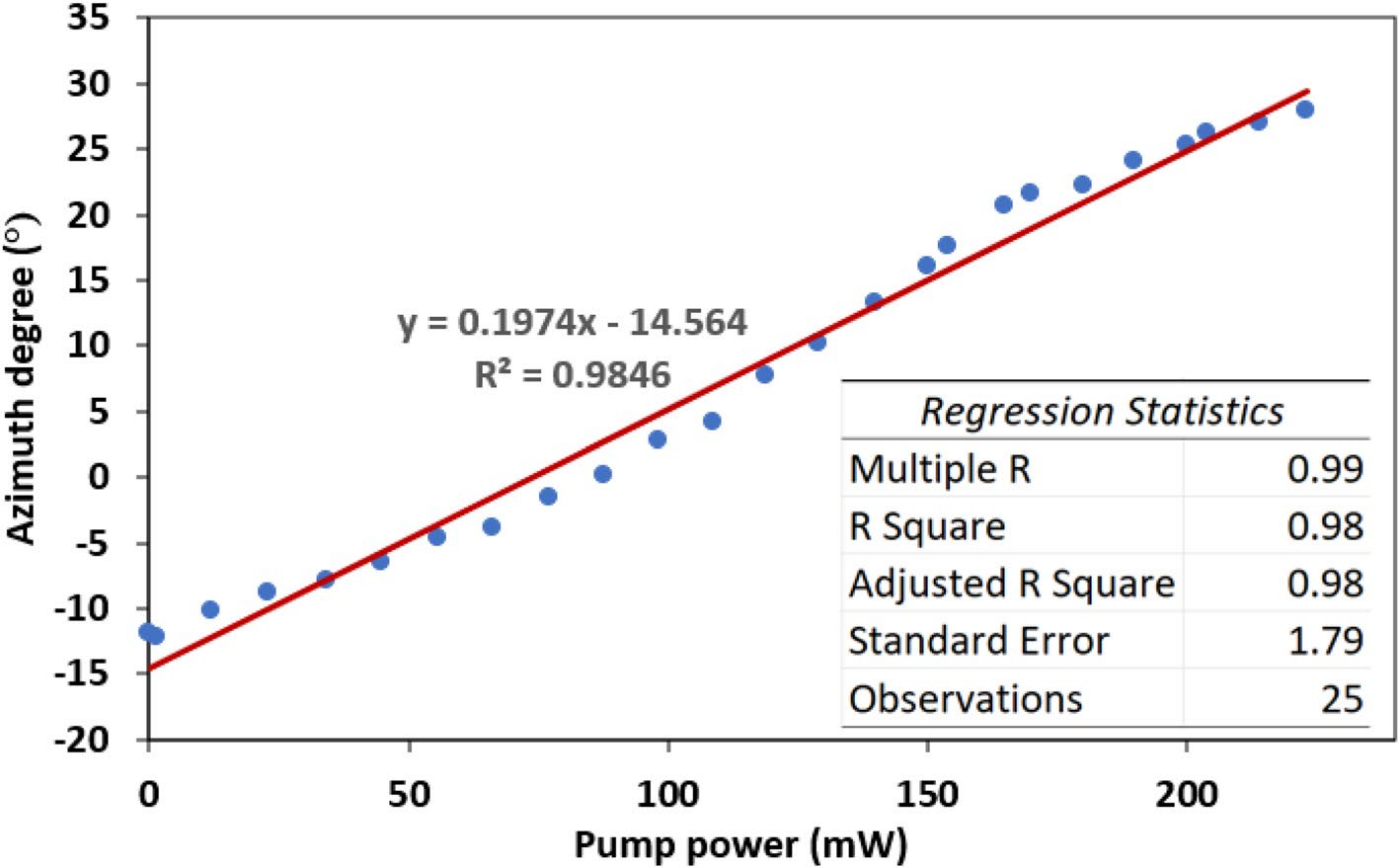
Rotation of light azimuth versus pump power.

Figure 7 shows the relationship between light azimuth and pump power. Light azimuth rotates from − 11.94° to 27.87°, translating to maximum rotation of 39.81°. From the regression analysis, the average azimuth rotation is ascertained at 0.1974°/mW. The R^2^ value of 0.9846 indicated the experimental data is well fitted with the linear regression equation of $y = 0.1974x - 14.564$. From the linear regression trend, there is no indication that the polarization rotation experience saturation with the increase of pump power, so the light azimuth can be further rotated if higher pump power is employed. When MXene absorbs continuous wave (CW) pump laser, it induces oscillation of electron from occupied state to unoccupied state and creates hot electrons that leads to thermal charge carrier distribution. Then, the hot electron cools down through transferring energy to the lattice phonon which results in a temperature increase^35,36^ The heat generated from the increment of pump power propagates through Nb_2_C thin film and modifies its refractive index due thermo-optic effect, which can be expressed by

1 $$n\left( T \right) = n_{0} \left( {T_{0} } \right) + \frac{dn}{{dT}}\Delta T$$

where ${\text{n}}_{{0}}$ is the refractive index at temperature $T_{0}$, $\frac{dn}{{dT}}$ is the thermo-optic coefficient and ${\Delta }T$ is the change rate of temperature. As a result, the propagation path of light changes, which leads to change of phase, and subsequently modulates the polarization state of signal^37,38^.

**Figure 8:**
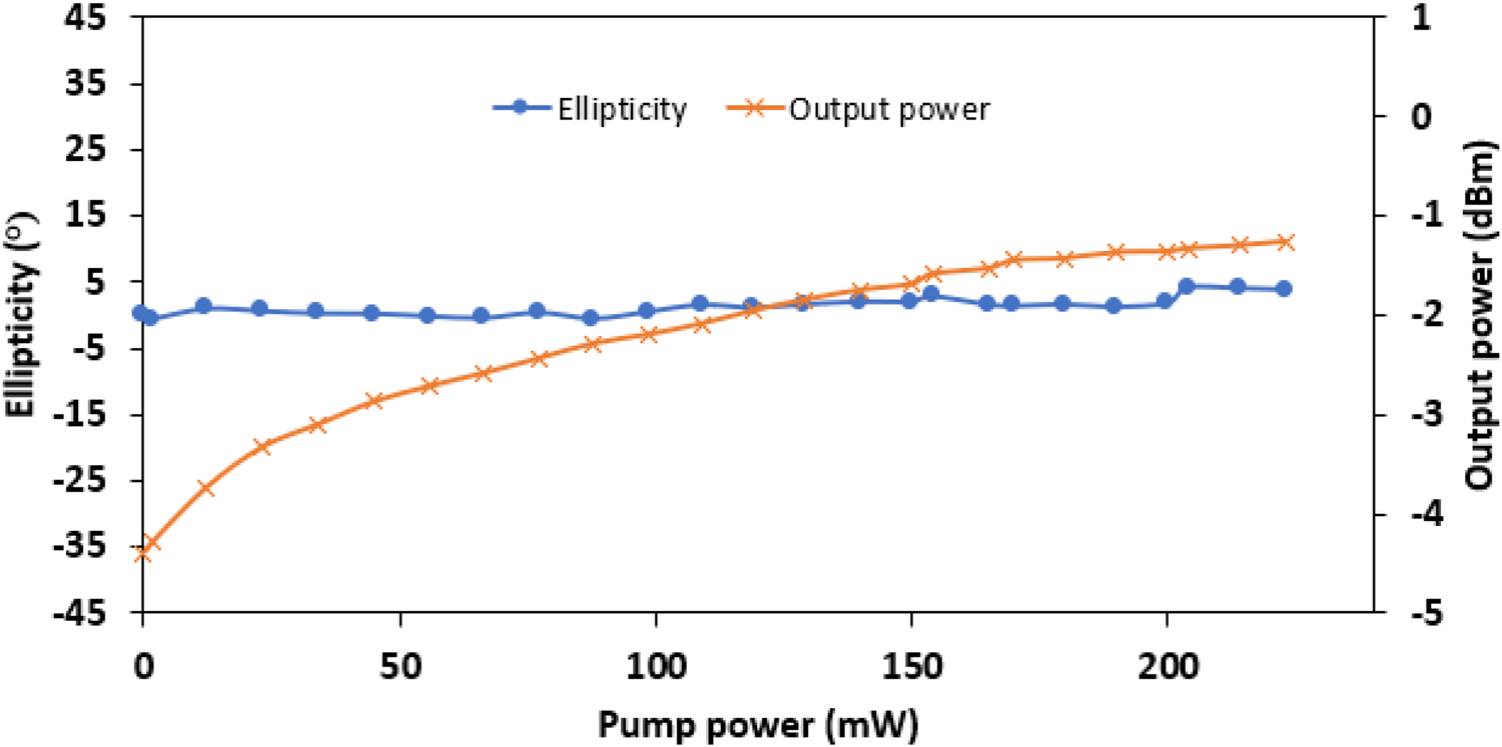
Light ellipticity and output power versus pump power.

The change of light ellipticity and output power with increment of pump power are illustrated in Fig. 8. No particular trend is discovered on the light ellipticity, but it fluctuates between 0.06° and 4.08° as the pump power is increased from 0 to 223 mW. On the other hand, the measured output power is increasing with pump power. The output power increased from − 4.64 to − 1.27 dBm throughout the increment of pump power. The increase of pump power feeding the absorption of the Nb_2_C thin film. As a result, the absorption of the Nb_2_C thin film reduced with the increase of pump power, allows the signal light experiences higher transmissivity. Table 1 shows the comparison of optical modulator that were demonstrated using various polarizing materials. In year 2015, Kan et al. has reported the polarization modulation with the employment of vertically deformable micro-electro-mechanical system to achieve 28° polarization rotation. In year 2017, Nicholls et al. reported polarization modulation using high intensity ultrafast laser as controller. There are some works reported two polarization state switching using MoS_2_^37^and plasmonic material^38^, but not able to perform fine tuning in between the high-Low state. It is worth to note that this work reveals higher modulation resolution compared to^16^ and the fluctuation of ellipticity is smaller (4.08°) compared to earlier work where the reported fluctuation was 10°.

**Table 1:** Comparison of polarization modulator with different polarizing materials.

Polarizing material	Wavelength	Total rotation	Resolution	References
MEMs chiral metamaterial	166.7–750 μm	28°	28°/deformation range	^39^
Anisotropic nonlinear metamaterial	700 nm	65°	1.67°/GW cm^−2^	^40^
2D material, MoS_2_ thin film	1550 nm	2 states	–	^37^
Plasmonic material, CDO thin film	2.08 μm	2 states, 65°	–	^41^
2D material, MoS_2_ thin film	1310 nm	70.81°	0.1304°/mW	^16^
2D material, NB_2_C thin film	1500 nm	39.82°	0.1974°/mW	This work

This work has proven the potential of MXene thin film to function as a linear polarization modulator. The proposed work is an alternative solution to compensate light polarization in an optical network by simply controlling the pump power. This set up can be installed along optical fiber to achieve acceptable polarization dispersion and bit error rate (BER). Therefore, the realization of long distance and high-speed optical network for future communication is feasible with this solution.

## Supplementary Information

Supplementary Information.

## Data Availability

All data generated or analysed during this study are included in this published article [and its supplementary information files].
